# Soft Biomimetic Fish Robot Made of Dielectric Elastomer Actuators

**DOI:** 10.1089/soro.2017.0062

**Published:** 2018-08-01

**Authors:** Jun Shintake, Vito Cacucciolo, Herbert Shea, Dario Floreano

**Affiliations:** ^1^ Institute of Microengineering Lausanne Campus, School of Engineering, Station 17, École Polytechnique Fédérale de Lausanne, Lausanne, Switzerland.; ^2^ Institute of Microengineering Neuchâtel Campus, School of Engineering, École Polytechnique Fédérale de Lausanne, Neuchâtel, Switzerland.

**Keywords:** underwater robots, fish robots, dielectric elastomer actuators, swimming robots

## Abstract

This article presents the design, fabrication, and characterization of a soft biomimetic robotic fish based on dielectric elastomer actuators (DEAs) that swims by body and/or caudal fin (BCF) propulsion. BCF is a promising locomotion mechanism that potentially offers swimming at higher speeds and acceleration rates, and efficient locomotion. The robot consists of laminated silicone layers wherein two DEAs are used in an antagonistic configuration, generating undulating fish-like motion. The design of the robot is guided by a mathematical model based on the Euler–Bernoulli beam theory and takes account of the nonuniform geometry of the robot and of the hydrodynamic effect of water. The modeling results were compared with the experimental results obtained from the fish robot with a total length of 150 mm, a thickness of 0.75 mm, and weight of 4.4 g. We observed that the frequency peaks in the measured thrust force produced by the robot are similar to the natural frequencies computed by the model. The peak swimming speed of the robot was 37.2 mm/s (0.25 body length/s) at 0.75 Hz. We also observed that the modal shape of the robot at this frequency corresponds to the first natural mode. The swimming of the robot resembles real fish and displays a Strouhal number very close to those of living fish. These results suggest the high potential of DEA-based underwater robots relying on BCF propulsion, and applicability of our design and fabrication methods.

## Introduction

As an emerging field, soft robotics has been the focus of major research efforts.^1,2^ Soft robots, that is, robots composed of compliant materials, offer important advantages over conventional rigid robots, such as simplified body structure and control,^3,4^ together with high robustness and versatility.^5,6^

One promising application of soft robotics is biomimetic underwater robots, wherein the high mobility and efficiency of aquatic animals could be achieved,^7^ by approximating their natural movements with the theoretically infinite number of degrees of freedom offered by soft-bodied robots. In addition to underwater applications such as inspection and environmental monitoring, biomimetic underwater robots could also serve as a platform to address biological questions related to the biomechanics and control of living fish.^8–10^ Within this context, researchers have recently developed soft underwater robots based on different actuation technologies, such as, ionic polymer–metal composites, lead zirconate titanate, shape memory alloys, fluidic elastomer actuators, and dielectric elastomer actuators (DEAs).^11–16^

Among these soft actuation technologies, DEAs^17–19^ show promising features for biomimetic underwater robots. DEAs are compliant (typical elastic modulus of ∼1 MPa), fast (response time <200 μs with suitable material choice^20^), efficient (theoretically maximum 90% of electromechanical efficiency^17^), and exhibit large actuation strokes (>85% of linear strain^21^). When immersed in water, dielectric elastomers show very little water absorption (up to 3.5% of own weight in 365 days^22^). In DEA devices, it has been reported that a cell stretcher interfacing liquid can function 24 h,^23^ and an underwater robot can swim >3 h.^14^ The latter consists of integrated power source and controller, demonstrating feasibility of self-contained DEA underwater robots. DEAs consist of a dielectric elastomer membrane sandwiched between two compliant electrodes. The application of high voltage (typically >1 kV) induces opposite charges on the electrodes, resulting in an electrostatic attractive force (Maxwell pressure), which squeezes the elastomer membrane in the thickness direction and generates an area expansion.

Based on DEAs, researchers developed a jellyfish robot,^13^ a ray robot,^14^ and a bimorph swimmer.^15^ We focus in this article on a fish-shaped robot, consisting of a body and a caudal fin, as one morphology of DEA-based underwater robots. Fish swimming is mainly divided into two types: body and/or caudal fin (BCF) propulsion and median and/or paired fin (MPF) propulsion.^7^ Although MPF locomotion offers maneuvering and stabilization, BCF locomotion enables swimming at higher speeds and acceleration rates with the most efficient movement (specifically, in case of thunniform mode). Therefore, employment of BCF locomotion, that is, a body and a caudal fin, can be a promising design approach for DEA-based underwater robots wherein high mobility and efficiency are expected. Also, given the diverse morphologies of fish and the fact that most of them generate thrust by BCF propulsion, the robots employing such a swimming mechanism could benefit from more design flexibility in terms of geometries and sizes. However, development of DEA-based underwater robots with BCF propulsion has not been attempted yet. For this reason, their designing principle, fabrication method, and performance characteristics are missing.

In this article, we report a model, fabrication method, and characterization of a DEA-based BCF fish robot consisting of a body and a caudal fin. This work is an expansion of a preliminary conference article,^24^ where first DEA-based swimming robots have been presented. In this article, we included a mathematical model, based on the Euler–Bernoulli beam theory, for predicting the natural frequencies of the robot in water, from which we can set the range of driving frequencies. Since the beating amplitude of the robot is comparable with the width of its body, we used a model able to describe large deformations. To validate the model, the outputs are compared with the characterization results of the fabricated robot. The fabrication process, which consists in laminating the silicone layers enabling the insulation of the high-voltage electrodes, is based on authors' preliminary results.^24^ In the current robot design, we reduced the number of layers from 5 to 4 by shaping each layer with the same geometry. The robot is characterized with fixed-free boundary conditions (the head is cramped whereas the tail is free to move) as well as tethered free-swimming condition. In the fixed condition, the tail amplitude and thrust force are measured. As for the tethered free-swimming condition, the swimming speed is measured and the Strouhal number is estimated. We observe that the model shows natural frequencies similar to the peaks of the measured thrust force and swimming speed. We also observe that the swimming locomotion of our robot resembles to nature; a Strouhal number very close to that of real fish represents it quantitatively.

## Materials and Methods

### Structure and swimming mechanism of the robot

**FIG. 1.**
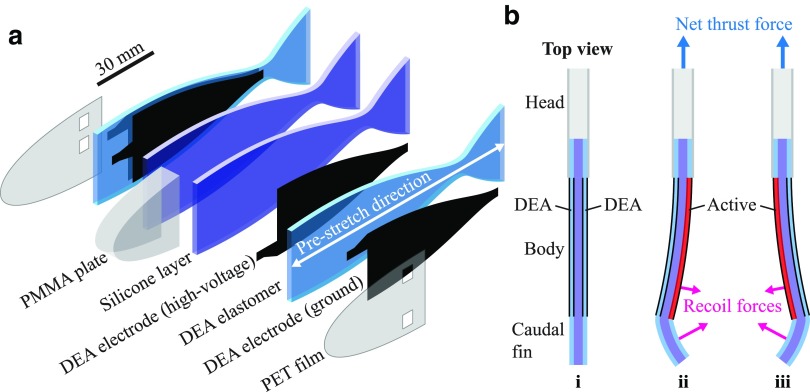
Structure of the fish robot and actuation principle. **(a)** The soft fish robot consists of four laminated silicone elastomer layers, forming an antagonistic configuration. **(b)** (i) The unactuated robot shape is straight due to the two DEAs that are equally prestretched. (ii, iii) When actuating each DEA periodically and sequentially, the recoil forces on the body and the tail lead to net thrust pushing the robot forward. DEAs, dielectric elastomer actuators. Color images available online at www.liebertpub.com/soro

The structure of the soft fish robot consists of four silicone elastomer layers that are laminated: two uniaxially prestretched DEAs sandwiching a body made of two silicone layers, forming an antagonistic configuration as shown in Figure 1a. The head part of the robot is made of a poly(methyl methacrylate) (PMMA) plate and two polyethylene terephthalate (PET) films. In this configuration, the high-voltage DEA electrodes are encapsulated between the silicone layers and the DEA elastomers, and are electrically insulated. The DEA electrodes on the groundside are instead exposed to the surrounding water. Thanks to this feature, the robot structure and fabrication process have been simplified, while enabling the analytical modeling of its dynamics, as described in the next section.

Figure 1b(i) shows a top view of the robot in nonactivated state. The robot shape is straight due to the equal prestretch in the two DEAs. In the robot, as also schematically represented in Figure 1a, DEAs are placed only on the body while there is no actuation part on the caudal fin, so that the latter passively deforms like that of real fish. When the DEA on one side is activated (Fig. 1b(ii)), it releases the internal stress of the prestretch and elongates, whereas the other one contracts, resulting in a global bending motion of the body. The body contraction moves the caudal fin that is deformed by the reaction force of the surrounding water. The recoil forces on the body and the caudal fin lead to net thrust pushing the robot forward. By actuating each DEA periodically (Fig. 1b(ii, iii)), the robot continuously generates the thrust force, leading to steady swimming in the forward direction.

### Model and design

Researchers experimentally have shown that an efficient thrust performance occurs around the first resonant frequency.^25,26^ Therefore, designing the soft fish robot addressing the matching between the structural natural frequency and the range of driving frequencies can be a reasonable approach. Moreover, several works have been done in robotics, exploiting the natural modes of vibration of the robotic structure to mimic fish-like swimming motions.^27,28^ Specifically, the work presented by El Daou *et al.*^28^ employed the second vibration mode.

**FIG. 2.**
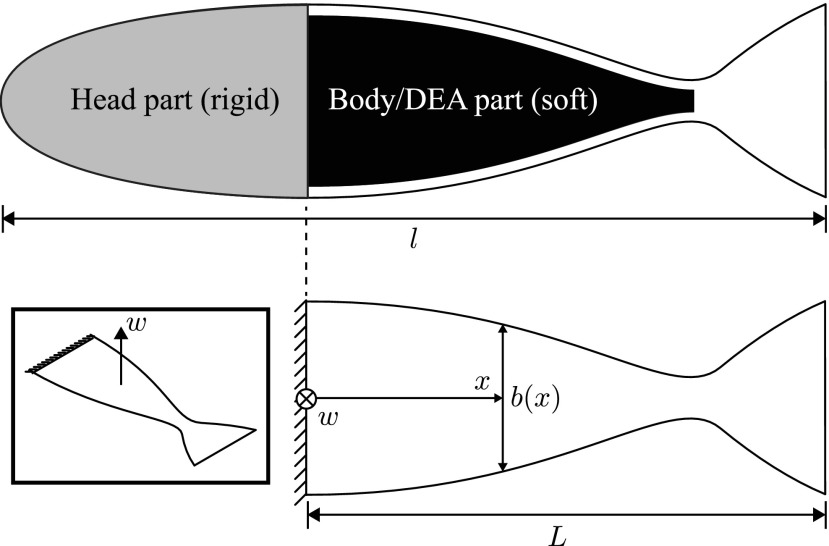
Schematics of the model that extracts the natural frequencies of the robot, by considering out-of-plane displacement of the body/DEA part *w* (*inset*). The rigid head part is assumed to be clamped. In these schematics, *l* is the total length of the robot, *L* the length of the body/DEA part, and *b* is the variable width as a function of the position in the longitudinal direction *x*.

In this context, the mathematical modeling of the fish robot in this study serves to extract the natural frequencies of the structure as a function of the design parameters, to match the chosen driving frequency range. As shown in Figure 2, the fish is modeled as a beam of constant thickness *h* and variable width $b \left( x \right)$, where *x* is the coordinate along the longitudinal axis of the body. The geometry of the robot is inspired by the profile of a trout and we used a formula proposed in Ref.^29^ to compute the target shape given the design parameters; we obtained $b \left( x \right) = 2 \left[ {{B_1} \sin ( {B_2}x ) + {B_3} \sin \left( {{e^{{B_4}x}} - 1} \right) } \right]$, where ${B_1} = 0.1l$, ${B_2} = 2 \pi / 1.4l$, ${B_3} = 0.00075l$, and ${B_4} = 2 \pi / 1.121l$, where *l* is the total length of the robot. In this study, we set *l* as 150 mm. As for the target range of the structural natural frequencies, we considered a driving frequency range from 0 to 3 Hz, as trout of length similar to *l* show steady swimming in this range.^30^ We describe the deformation of the structure through the Euler–Bernoulli beam theory.^31^

\begin{align*}
{ \left[ {K \left( x \right) \ w \prime \prime \left( {x , t} \right) } \right] ^{ \prime \prime }} + { \varrho _s} \left( x \right) \ \ddot w \left( {x , t} \right) = H \left( {x , t} \right) + s \left( {x , t} \right) , \tag{1}
\end{align*}

where $K \left( x \right)$ is the bending stiffness, $w \left( {x , t} \right)$ is the out-of-plane displacement, ${ \varrho _s} \left( x \right)$ is the mass density per unit length, $H \left( {x , t} \right)$ is the function of hydrodynamic forces, and $s \left( {x , t} \right)$ represents the structural damping of the body. In addition, dots $\cdot$ refer to differentiation with respect to time *t*, whereas apices $\prime$ refer to differentiation with respect to *x*. Equation (1) is a partial differential equation (PDE) with nonconstant coefficients and it is valid to describe the deformation of the fish if we have $L \ll b \left( x \right) \ll h$, where *L* is the length of the structure. The coefficients ${ \varrho _s} \left( x \right)$ and $K \left( x \right)$ vary with *x* due to the nonconstant with $b \left( x \right)$. As for the mass density, we define it as

\begin{align*}
{ \varrho _s} \left( x \right) = 2 \ b \left( x \right) \ \rho \left[ {{h_{BODY}} + {h_{DEA}}} \right] , \tag{2}
\end{align*}

where $\rho$ is the density of the solid material, in this case a silicone elastomer, whereas ${h_{BODY}}$ and ${h_{DEA}}$ are the thicknesses of the body silicone layers and the DEA layers, respectively. To simplify the model, we neglect the mechanical effect of the electrode layers due to their thickness being much smaller than the other layers. We also neglect the prestretch of the DEAs because of their low ratio and equilibrium configuration. As for the stiffness, we accounted for the different Young's moduli between the body layers and the DEA layers using the method developed by Timoshenko,^32^ which consists in using an equivalent cross section with a homogeneous Young's modulus, correspondent to the higher one (in our case the body layers), where the width of the layer with lower Young's modulus is virtually reduced to account for the minor stiffness, according to

\begin{align*}
 { b_ { DEA } } \left( x \right) = b \left( x \right) { \frac { { E_ { DEA } } }  { { E_ { BODY } } } } . \tag { 3 } 
\end{align*}

So, the resulting stiffness can be computed as

**Figure:**
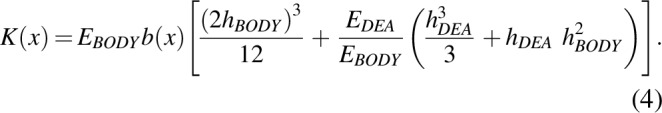

We model the oscillations of the fish with its head clamped, so the beam becomes a cantilever with fixed-free boundary conditions, that is

\begin{align*}
\begin{split}& w \left( {0 , t} \right) = 0 , \ w \prime \left( {0
, t} \right) = 0 , \ \\  & K \left( L \right) w \prime \prime
\left( {L , t} \right) = 0 , { \left. {{{ \left[ {K \left( x
\right) w \prime \prime \left( {x , t} \right) } \right] }^{
\prime \prime }}} \right\vert _{x = L}} = 0.\end{split}
 \tag{5}
\end{align*}

Following the methodology proposed by Aureli *et al.*,^33^ we can rewrite Equation (1) in frequency domain as

\begin{align*}
\frac{{\left( {1 + i\eta } \right)}}{{{\varrho _s}\left( x
\right)}}{\left[ {K\left( {{x}} \right)\;{{{{\hat w}}}^{\prime
\prime }}\left( {{{x}},{{\omega }}} \right)} \right]^{\prime
\prime }} - {{{\omega }}^2}\;{{\hat w}}\left( {{{x}},{{\omega }}}
\right) = ,\\ {{ = }}{{{\omega }}^2}\;{{M}}\left( {{x}}
\right)\;\Theta \left( {{{\beta }}\left( {{{x, \omega }}}
\right){{, k}}\left( {{{x , \omega }}} \right)} \right)\;{{\hat
w}}\left( {{{x, \omega }}} \right)\tag{6}
\end{align*}

where $M \left( x \right) = { \frac { \pi { \rho _f } b { { \left( x \right) } ^2 } }  { 4 \ { \varrho _s } \left( x \right) } }$ is the nondimensional ratio between the mass densities of the fluid, in this case water, and the solid; $\Theta$ is the complex nondimensional hydrodynamic function, which depends on the frequency parameter $\beta \left( { x , \omega } \right) = { \frac { { \rho _f } \omega b { { \left( x \right) } ^2 } }  { 2 \pi \mu } }$ and on the local Keulegan–Carpenter number $k \left( x \right) = { \frac { 2 \pi }  { b \left( x \right) } } \left\vert { \hat { w } \left( { x , \omega } \right) } \right\vert$; $\mu$ is the dynamic viscosity of the surrounding fluid, i.e., water.

For the scope of this work, the model has to only estimate the natural frequencies of the fish robot swimming in water in clamped mode. For this reason, we decided not to solve the full Equation (6) by including the hydrodynamic functions and damping effects, which would result in additional mathematical complexity that lies outside the scope of this article. We instead first of all extract the natural frequencies of the beam in vacuum. We then use the well-known inviscid approximation proposed by Sader to compute the correspondent natural frequencies in water^34^:

\begin{align*}
 { \frac { { f_f } }  { { f_v } } } = { \left( { 1 + { \frac { \pi { \rho _f } \bar { b } }  { 4 \ \rho h } } } \right)^ { - 1 / 2 } } , \tag { 7 } 
\end{align*}

where ${ \rho _f}$ is the density of the fluid. Equation (7) is derived for beams with constant width *b*, so we approximate it by using a reference value 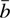 extracted from $$b \left( x \right)$$.

The modal analysis in vacuum for the undamped beam is conducted by taking Equation (6) with the boundary conditions Equation (5) and letting $\Theta \left( { \beta \left( {x , \omega } \right) , k \left( {x , \omega } \right) } \right) = 0$, $\eta = 0$:

\begin{align*}
 \frac { 1 }  { { { \varrho _s } \left( x \right) } } { \left[ { K \left( x \right) { { \hat { w } } } ^ { \prime \prime } } \left( { x , \omega } \right) \right] } ^ { \prime \prime } - { \omega ^2 } \ \hat { w } \left( { x , \omega } \right) = 0. \tag { 8 } 
\end{align*}

Even with these assumptions, due to the variable width $b \left( x \right)$, both ${ \varrho _s} \left( x \right)$ and $K \left( x \right)$ are not constant with *x* and even nonlinear functions of *x*, so the resulting PDE (8) cannot be solved analytically. We chose to apply the Galerkin method, projecting the solutions of Equation (8) on the vibration modes $\left\{  {{ \phi _i} \left( x \right) } \right\} _{i = 1}^m$ of a rectangular cantilever beam with uniform width in vacuum, which are^31,33^

**Figure:**
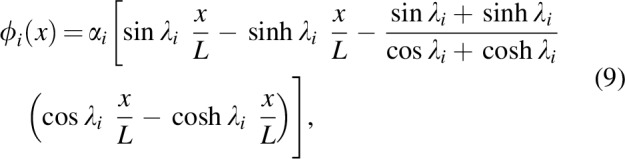

where ${ \alpha _i}$ are scaling factors that guarantee that

\begin{align*}
\frac{1}{L}\int\limits_0^L {{\phi _i}\left( x \right){\phi
_j}\left( x \right)dx = {\delta _i}_j} {\rm{ }} \tag { 10 }
\end{align*}

and ${ \lambda _i}$ are the solutions of the characteristic equation cos λ_*i*_ cosh λ_*i*_ + 1 = 0. The first few values are λ_1_ = 1.875, λ_2_ = 4.694, λ_3_ = 7.855 and ${ \alpha _1} = 1.36 , \ { \alpha _2} = 0.98 , \ { \alpha _3} = 1.00$.

The projection of Equation (8) on the eigenfunctions $\left\{  {{ \phi _i} \left( x \right) } \right\} _{i = 1}^m$ consists in approximating the deflection as

\begin{align*}
 \hat{w} \left( {x , \omega } \right) = \mathop \sum \limits_{j = 1}^ \infty {q_j} \left( \omega \right) { \phi _j} \left( x \right) , \tag{11}
\end{align*}

multiplying by ${ \phi _i} \left( x \right)$ and integrating in the domain $x \in \left[ {0 , L} \right]$, which in matrix form can be written as

\begin{align*}
\chi \;q\left( \omega  \right) - {\omega ^2}{\rm{ }}\Psi \;q\left(
\omega  \right) = 0. \tag{12}
\end{align*}

With

\begin{align*}
{\chi _{ij}} = \frac{1}{L}\int\limits_0^L {{\phi _i}\left( x
\right)\frac{1}{{{{\tilde n}_s}\left( x \right)}}\left[ {K\left( x
\right)\phi _j^{\prime \prime }\left( x \right)} \right]\prime
\prime dx} \tag { 13 }
\end{align*}

\begin{align*}
{\Psi _{ij}} = \frac{1}{L}\int\limits_0^L {{\phi _i}\left( x
\right){\phi _j}\left( x \right)dx = {\delta _{ij}} \to {\rm{
}}\Psi  = {I_m},} \tag { 14 }
\end{align*}

where ${q_i} \left( \omega \right)$ are the weights correspondent to the $i{ \rm{th}}$ mode ${ \phi _i} \left( x \right)$. Equation (12) represents an eigenvalue problem. The eigenvalues $\omega$ of $\boldsymbol{\chi}$ are the natural frequencies of the undamped fish in vacuum, whereas the eigenvectors $\textbf{\textit{q}} \left( \omega \right)$ are the correspondent sets of weights. We computed numerically all the integrals in ${ \chi _{ij}}$ by choosing the number of shape functions for the projection of the solution as $m = 10$. The first six natural frequencies in vacuum obtained from the model are given in Table 1, and the specification of the robot and material parameters used are summarized in Table 2.

**Table 1.** Natural Frequencies of the Robot Structure in Vacuum Obtained from the Model

*i*	*1*	*2*	*3*	*4*	*5*	*6*
${f_{vi}}$ [Hz]	0.47	2.32	6.43	12.67	21.35	32.15

**Table 2.** Specification and Materials of the Robot

*Design parameter*	*Value*
Dimensions	
Total length of the robot *l*	150 mm
Length of the robot excluding the head part *L*	110 mm
Maximum width ${b_{max}}$ ($x = 0$ mm)	35 mm
Minimum width ${b_{min}}$ (($x = 70$ mm)	6 mm
Total thickness of the robot *h*	700 μm
DEA layer thickness ${h_{DEA}}$	100 μm
Body layer thickness ${h_{BODY}}$	250 μm
Material property	
Young's modulus of the DEA layer ${E_{DEA}}$	0.83 MPa^39^
Young's modulus of the body layer ${E_{BODY}}$	2.0 MPa^40^
Density of the silicone layers $\rho$^a^	1070 kg/m^3^
Density of water ${ \rho _f}$	998.23 kg/m^3^
Dynamic viscosity of water $\mu$	8.90 × 10^−4^ Pa·s
Other	
Prestretch ratio of the DEAs	1.25

^a^ Value is average of the silicones used: 1110 kg/m^3^ for the DEA layer and 1030 kg/m^3^ for the body layer.

DEAs, dielectric elastomer actuators.

As for the computation of the natural frequencies in water, from the theory on underwater vibrations of beams, we expect the values of the frequencies to decrease due to the hydrodynamic added mass of water. As shown by Sader's formula (7), the ratio between natural frequencies in fluid and natural frequencies in vacuum depends on the ratio between the densities of the fluid and densities of solid materials. Considering that in our case we used silicone elastomer, whose density is very close to that of the fluid, we expected a high decrease of natural frequencies in water for our fish robot. Sader's formula is an inviscid approximation, which is reliable in case the oscillatory Reynold's number $Re = \pi { \rho _f}f{b^2} / 2 \mu$ is $Re \gg 1$. Usually for beams in transverse vibration, the reference length used in *Re* is the width, which in our case is variable ($b = b \left( x \right)$), so we can define ${b_{min}} = 6 \ { \rm{mm}}$ and ${b_{max}} = 35 \ { \rm{mm}}$. By using as extremes of our range of vibration ${f_{v1}} = 0.47 \ { \rm{Hz}}$ and ${f_{v6}} = 32.15 \ { \rm{Hz}}$, we can compute the two extremes $Re \left( {{b_{min}} , {f_{v1}}} \right) = 26.50$ and $Re \left( {{b_{max}} , {f_{v6}}} \right) = 61.74 \cdot {10^3}$, from which we can see that the hypothesis of inviscid fluid is satisfied. Therefore, we used Equation (7) to estimate the natural frequencies in water. From empirical observations, we chose to set as reference width $\overline b$ in Equation (7) the minimum width, so $\overline b = {b_{min}}$ and we obtained the natural frequencies in water given in Table 3.

**Table 3.** Natural Frequencies of the Robot Structure in Water Obtained from the Model (Fixed-Free Boundary Condition)

*i*	*1*	*2*	*3*	*4*	*5*	*6*
${f_{fi}}$ (Hz)	0.17	0.83	2.31	4.56	7.68	11.56

### Fabrication

**FIG. 3.**
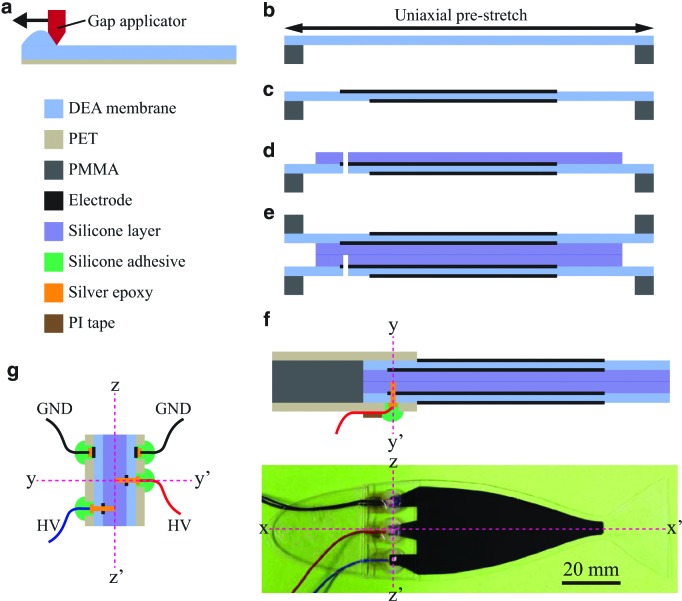
Fabrication process of the robot. **(a)** DEA elastomer is blade casted on a PET film and **(b)** stretched uniaxially. **(c)** Electrodes are patterned on the stretched membrane. **(d)** Body layer made of silicone elastomer is bonded and a punched hole is made. **(e)** Bonding two half samples. **(f)** Attaching the head part and wiring. **(g)** Alignment of the electrode connections. PET, polyethylene terephthalate. Color images available online at www.liebertpub.com/soro

The fabrication process of the robot is mainly divided into four steps: casting silicone elastomer layers, patterning electrodes, bonding of the silicone layers, and wiring of electrical connections. Figure 3a–f shows the fabrication steps. In this study, two different silicone elastomers were used: Nusil CF19-2186 and Dow Corning Sylgard 184. The former was used for the DEAs and the latter was used for the robot body. First, the silicone CF19-2186 was mixed with the manufacturer-recommended ratio for 1 min at 2000 rpm using a planetary mixer (Thinky ARE-250). The uncured silicone mixture was blade casted on a PET film using an applicator coater (Zehntner ZUA2000) and variable gap applicator (Zehntner ZAA2300), and cured in oven at 80°C for 1 h. After curing, the DEA membrane (thickness of ∼100 μm) was separated from the PET film and suspended in a PMMA frame with a silicone adhesive foil (Adhesives Research ARclear 8932EE) while being stretched uniaxially with a ratio of 1.25. Subsequently, electrodes made of a mixture of carbon black and soft silicone were patterned on both sides of the DEA membrane, using the pad-printing method. The details of the electrode composition and the pad printing are available in the literature.^35^ After the patterning of the electrodes, the robot body layer, with a thickness of ∼250 μm, was chemically bonded to the DEA, using oxygen plasma surface activation (Diener electronic Zepto plasma system); the insertion of ethanol droplets in the bonding interface helps in removing air bubbles.^36^ Once the DEA was fully bonded to the body layer, a hole was punched for the electrical connection. This sample was then again chemically bonded to another sample, which has different electrode shape, so that the connections of the high-voltage electrodes of the two DEAs do not overlap each other. Figure 3g shows details of the alignment of the electrodes and the connections. The entire part was then cut off from the frame with the desired shape, followed by attaching the head parts consisting of a PMMA plate and the PET film. Finally, the wiring was made using a conductive silver epoxy (Amepox ELECTON 40AC), polyimide tape, and a liquid silicone adhesive (Dow Corning Sylgard RTV-734). The mass of the assembled robot is 4.4 g.

### Experimental setup

The fabricated robot was characterized in both fixed and tethered swimming conditions. All the characterizations were performed in a water tank with dimensions of 50 cm (*L*) × 40 cm (*W*) × 12 cm (*H*), filled with tap water. The robot was activated through a high-voltage converter (EMCO Q50) and a microcontroller board generating high-voltage sine waves. The range of voltage and frequency used in this study was 0–5 kV and 0–3 Hz, respectively. In the fixed swimming condition, the head part of the robot was mounted to a load cell (Applied Measurement Limited UF1) to measure the thrust force, and to a PMMA plate to observe the tail amplitude. The tail amplitude refers to the peak-to-peak displacement of the tip of the caudal fin in steady state oscillation. A CMOS camera was used to record the actuated deformations of the robot to assess the tail amplitude by image processing. The thrust force was measured by averaging the sensor value for 10 s. In the tethered swimming condition, the swimming speed was measured using a CMOS camera and a scale. Each measurement was repeated three times at every driving frequency or voltage step, and the average value was reported. Thin copper wires with a diameter of 36 μm were used to drive the robot to minimize the mechanical resistance during swimming.

## Results and Discussion

**FIG. 4.**
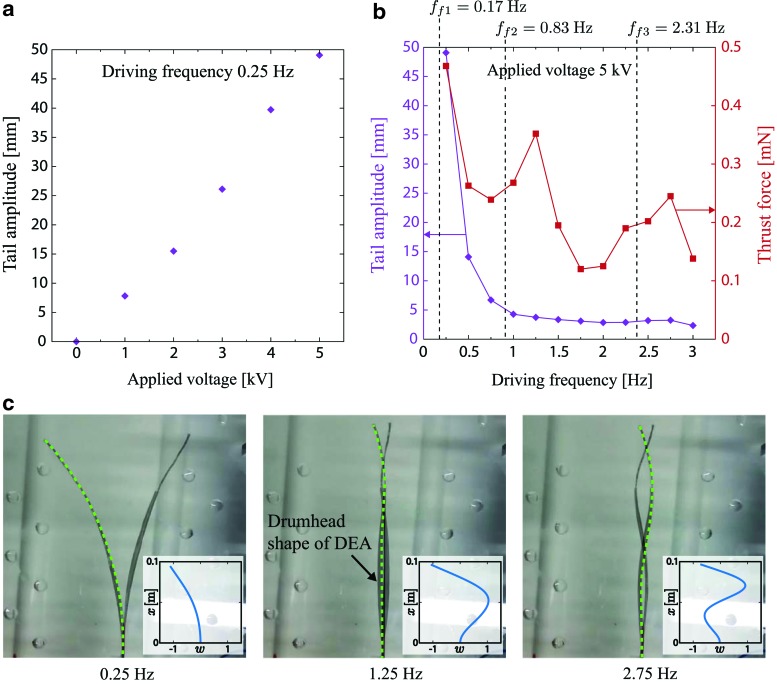
Characterization results of the robot with fixed-free boundary condition. **(a)** Measured tail amplitude as a function of the applied voltage, at the driving frequency of 0.25 Hz. **(b)** Measured tail amplitude and thrust force as functions of the driving frequency at the applied voltage of 5 kV. *f_f1-3_* are the natural frequencies computed with the model. **(c)** Deformations of the robot at the applied voltage of 5 kV. *Green lines* represent the topside of the robot structure. The *inset* graphs show simulated resonance mode shapes corresponding to the first (*left*), second (*middle*), and third (*right*) modes. The deformation w is scaled. Color images available online at www.liebertpub.com/soro

The tail amplitude as a function of the applied voltage at the driving frequency of 0.25 Hz is presented in Figure 4a. The amplitude increases almost linearly with the voltage, and a maximum amplitude of 49.1 mm is observed at 5 kV. Figure 4b shows the plots of the tail amplitude and the thrust force as functions of the driving frequency at the applied voltage of 5 kV. While the amplitude decreases smoothly with the frequency, the force shows a similar trend but peaks at 1.25 and 2.75 Hz, respectively. These peaks suggest the presence of resonance modes, and are visible in their shape at those corresponding frequencies, as shown in Figure 4c. In this figure, the inset graphs show simulated resonance mode shapes. At 1.25 Hz, the deformation of the robot is analogous to the second mode shape. Similarly, at 2.75 Hz, the third mode shape appears. The results also suggest that there would be the first mode whose shape is similar to that of 0.25 Hz, at a frequency around this value. These peak frequencies (0.25, 1.25, and 2.75 Hz) are close to the model result (0.17, 0.83, and 2.31 Hz) and take higher values, as indicated in Figure 4b. The difference between the model and experiments may be because of three main causes. First is the presence of the electrode layers that can make the bending stiffness of the structure higher and, therefore, increase the natural frequencies. The second is the stiffening of the silicone elastomers due to the oxidation by the oxygen plasma surface activation,^33^ which should again result in higher values of the frequencies. Finally, the third is the use of Sader's formula (7) to map the natural frequencies of the robot in vacuum to those in water introduces an additional source of error since the formula was derived for beams with constant width.

The tail amplitude does not show peaks at those frequencies. This suggests that the generation of the thrust force does not depend only on the amplitude of the tail beat, but rather on the whole body deformation, which shows a large excitation in correspondence to the resonance frequencies. The observations in real fishes support our sight that subcarangiform swimmers, that is, trout fishes from which we obtained the robot geometry, use half of their body to generate thrust force and not only the tail.^7^ The measured thrust force also shows a decreasing trend as the frequency is increased. A possible reason is the reduction of the tail amplitude toward higher frequency. One potential solution for compensating the force reduction is to implement a variable stiffness element made of phase change materials into the robot. Thanks to this element, the body stiffness could be modulated to shift the resonance frequencies, leading to larger tail amplitude and thrust force at higher frequencies. In the shapes of the robot presented in Figure 4c, especially that correspondent to 1.25 Hz, the body shows a drumhead shape, due to the nature of the DEAs that elongate also in the in-plane direction perpendicular to the longitudinal axis (i.e., head–tail axis). This phenomenon may be an additional reason for the discrepancy between the experimental data and the model, which does not include this effect. The drumhead shape may also have a negative influence on the thrust force. If so, one solution to prevent this effect would be to adjust the prestretch ratio of the DEAs. It is known that DEAs deform perpendicularly with respect to the direction of the prestretch.^38^ Therefore, prestretching of the DEAs also in the width direction can be beneficial. Therefore, prestretching of the DEAs also in the width direction can be beneficial.

**FIG. 5.**
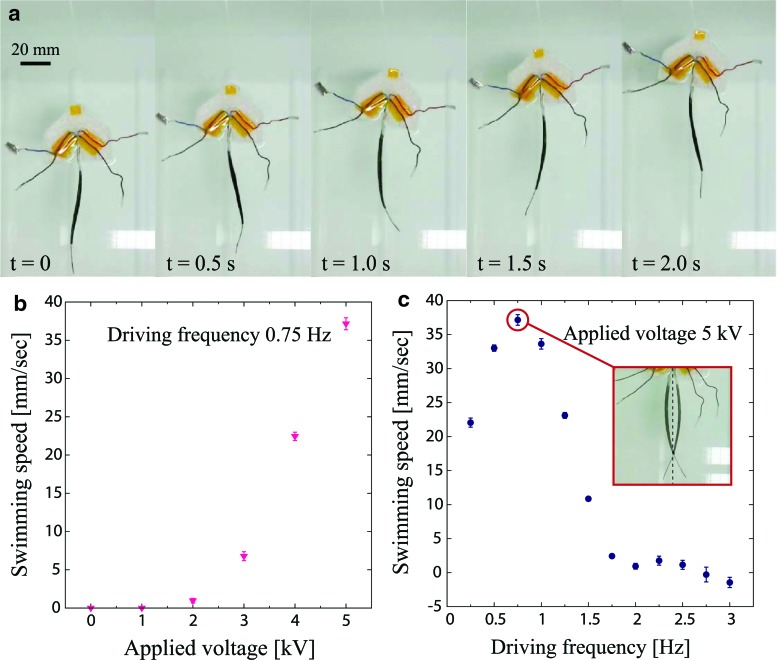
Characterization results of the robot with tethered free-swimming condition. **(a)** Swimming sequence of the robot for a driving frequency of 0.75 Hz at the applied voltage of 5 kV. **(b)** Measured swimming speed as a function of the applied voltage at the driving frequency of 0.75 Hz. **(c)** Measured swimming speed as a function of the driving frequency at the applied voltage of 5 kV. Color images available online at www.liebertpub.com/soro

Figure 5a shows a sequence of the robot swimming under the tethered condition at the driving frequency of 0.75 Hz with an applied voltage of 5 kV (see also Supplementary Video S1; Supplementary Data are available online at www.liebertpub.com/soro). We observed that the swimming motion exhibited by the robot resembles real fish. Figure 5b presents the swimming speed at 0.75 Hz as a function of the applied voltage. The swimming speed increases with the applied voltage. During swimming, the head of the robot is moving due to the recoil forces that create a moment about its center of mass. Therefore, unlike our assumption, the robot structure is no longer considered as a perfect cantilever in the tethered swimming condition. This is obvious in Figure 5a where the head of the robot is rotating. The power consumption of the robot is measured to be 0.92 W. However, this will be greatly reduced by using a powering strategy wherein electric charges on the DEA capacitors are collected at each cycle. Throughout the experiments, the robot did not experience dielectric breakdown. Yet, breakdown failure of the device would appear when applying a voltage beyond its breakdown strength or as a consequence of fabrication errors.

Figure 5c shows the swimming speed as a function of the driving frequency at the applied voltage of 5 kV. The swimming speed has a peak value of 37.2 mm/s (0.25 body length/s) at 0.75 Hz, and shows a trend different from the thrust force that has peaks at 1.25 and 2.75 Hz. The difference of the peak positions results from the change of boundary conditions that shifts the value of resonance frequencies. We assume that the first mode appears at 0.75 Hz, given the shape shown in Figure 5c inset, which is the same as that observed for the first natural frequency in the clamped configuration (Fig. 4c). In Figure 5c, interestingly the swimming speed takes a negative value at 3 Hz and the robot swims backward. This effect may also result from the boundary conditions, since the head assumes an amplitude larger than the tail at the corresponding vibration mode.

To compare the swimming of our robot with real fish, we estimate the Strouhal number defined as

\begin{align*}
St = \frac { { fA } }  { U } , \tag { 15 } 
\end{align*}

where *f* is the driving frequency, *A* is the tail amplitude, and *U* is the swimming speed. It is known that the swimming of various species of fish (thunniform, subcarangiform, and carangiform) corresponds to a Strouhal number in a specific range of 0.25 < *St* < 0.40.^7^ We found that the tail amplitude in the tethered swimming condition at 0.75 Hz to be 23.5 mm by estimating from Figure 5c inset, resulting in the Strouhal number of the robot to be *St* = 0.47, which is very close to the range already mentioned for real fish. However, it should be fair to mention that such a range of *St* is known to be valid in a range of Reynolds number *Re* between 10^4^ and 10^6^ (*Re* = *LU/ν*, where *L* is a characteristic length and *ν* is the kinematic viscosity of water). Our robot has *Re* of 5.6 × 10^3^, slightly lower than the range, so it is uncertain whether the *St* obtained is still valid.

## Conclusion and Future Work

We have presented modeling, designing, fabrication, and characterization of a fish type DEA-based soft biomimetic underwater robot that swims by BCF propulsion. The mathematical model used to compute the natural frequencies of the structure showed values similar to the experimental results. The robot exhibited swimming motion resembling real fish, as also quantitatively estimated by the Strouhal number. These results suggest that the high potential of DEA-based underwater robots relies on BCF propulsion and the applicability of our design and fabrication methods. Our future work will consist in expanding the mathematical model to the tethered swimming condition. Specifically, the model should not consider the robot head as a fixed boundary, but should represent it as a point mass with free boundary condition. In this future model, the stiffening effects from the presence of the electrode layers and the oxidation due to oxygen plasma bonding will also be included. Subsequently, we will work on characterizing robots in different size scales and swimming modes to understand how far our model and building method are applicable.

## Supplementary Material

Supplemental data
